# Supplementary material for: Unravelling Anti-Melanogenic Potency of Edible Mushrooms Laetiporus sulphureus and Agaricus silvaticus In Vivo Using the Zebrafish Model
Source: J Fungi (Basel). 2021 Oct 5;7(10):834. doi: 10.3390/jof7100834 (PMC8540621; doi:10.3390/jof7100834)
Supplement: Supplementary file 1 [file jof-07-00834-s001.zip › jof-1384855-supplementary.pdf]

Table S1. Lethal and teratogenic effects observed in zebrafish (*Danio rerio*) embryos at different hours post fertilization (hpf).

Category	Developmental Endpoints	Exposure Time (hpf)			
		**24**	**48**	**72**	**96/120**
Lethal effect	Coagulated eggs [a]	●	●	●	●
	Lack of the heart beating	●	●	●	●
Teratogenic effect	Malformation of head	●	●	●	●
	Malformation of eyes [b]	●	●	●	●
	Malformation of sacculi/otoliths [c]	●	●	●	●
	Malformation of chorda	●	●	●	●
	Malformation of tail [d]	●	●	●	●
	Scoliosis	●	●	●	●
	Yolk edema	●	●	●	●
	Liver necrosis			●	●
	Yolk deformation	●	●	●	●
	Growth retardation [e]		●	●	●
	Hatching			●	●
Cardiotoxicity	Pericardial edema		●	●	●
	Heart morphology			●	●
	Heart beating rate (beat/min)				●

[a] No clear organs structure is recognized. [b] Malformation of eyes was recorded for the retardation in eye development and abnormality in shape and size. [c] Presence of none, one or more than two otoliths per sacculus, as well as reduction and enlargement of otoliths and/or sacculi (otic vesicles). [d] Tail malformation was recorded when the tail was bent, twisted or shorter than to control embryos as assessed by optical comparation. [e] Growth retardation was recorded by comparing with the control embryos in a body length (after hatching, at and onwards 72 hpf) using by optical comparison using an inverted microscope (CKX41; Olympus, Tokyo, Japan).
